# Correction to: Flow of online misinformation during the peak of the COVID-19 pandemic in Italy

**DOI:** 10.1140/epjds/s13688-021-00296-5

**Published:** 2021-07-29

**Authors:** Guido Caldarelli, Rocco De Nicola, Marinella Petrocchi, Manuel Pratelli, Fabio Saracco

**Affiliations:** 1 Department of Molecular Sciences and Nanosystems, Ca’Foscari University of Venice, Ed. Alfa, Via Torino 155, 30170 Venezia Mestre, Italy; 2 European Centre for Living Technology (ECLT), Ca’ Bottacin, 3911 Dorsoduro Calle Crosera, 30123 Venice, Italy; 3 IMT School For Advanced Studies Lucca, Piazza San Francesco 19, 55100 Lucca, Italy; 4 Institute of Informatics and Telematics, National Research Council, via Moruzzi 1, 56124 Pisa, Italy; 5 CINI – National Laboratory for Cybersecurity, via Ariosto, 25, 00185 Roma, Italy

**Correction to:** EPJ Data Science (2021) 10:34, 10.1140/epjds/s13688-021-00289-4

Following publication of the original article [1], it was reported that the Supplementary Information contained text in magenta, which should be printed in black.

The original article [1] has been corrected.
